# The effect of *Imaginary Working Qigong* on the psychological well-being of college students: Study protocol for a randomized controlled trial

**DOI:** 10.1097/MD.0000000000013043

**Published:** 2018-11-02

**Authors:** Yu Guo, Mingmin Xu, Meiqi Ji, Zeren Wei, Jialei Zhang, Qingchuan Hu, Jian Yan, Yue Chen, Jiaxuan Lyu, Xiaoqian Shao, Ying Wang, Jiamei Guo, Yulong Wei

**Affiliations:** a School of Acupuncture-Moxibustion and Tuina, Beijing University of Chinese Medicine, Beijing; b Ovation Health Science and Technology Co. Ltd, ENN Group, Langfang; c School of Acupuncture-Moxibustion and Tuina, Chengdu University of Traditional Chinese Medicine, Chengdu; d Department of Ophthalmology, China-Japan Friendship Hospital, Beijing, China.

**Keywords:** college students, concrete thinking, Imaginary Working Qigong, psychological health, randomized controlled trial

## Abstract

**Introduction:**

College students are special populations that are particularly prone to have significantly high level of psychological distress than their community peers. Apparently, the best way to manage stress and mental state is through self-care. The characteristic of Qigong is self-directed and self-healing, which is a traditional Chinese mind-body exercise, which has the potential as a stress management intervention. *Imaginary Working Qigong*, as a kind of static Qigong, is more perception-oriented and can bring about benign sensations of mind and body so as to eliminate stress completely and induce physiological and mental relaxation. In this study protocol, we will systematically examine the feasibility and acceptability of *Imaginary Working Qigong* on psychological outcomes of the college students and deeply explore molecular biological mechanisms underlying the effects of mind adjustment induced *Imaginary Working Qigong*.

**Methods/designs:**

We will conduct a randomized, assessor and statistician-blinded, parallel-controlled trial exploring the beneficial mind adjustment of *Imaginary Working Qigong* in college students.

A total of 80 eligible college students from Beijing University of Chinese Medicine will be recruited and randomly allocated into *Imaginary Working Qigong* training or unaltered lifestyle control group according 1:1 allocation ratio with allocation concealment. *Imaginary Working Qigong* training will last 8 weeks. The study period is 12 weeks including a 4-week supervised training, 4-week independence training, and a 4-week follow-up. Relevant psychological outcomes measurement will take place at baseline, 5 weeks (at the end of supervised training), 9 weeks (at the end of independence training), and 13 weeks (after the 4-week follow-up period) by blinded independent outcome assessors.

**Conclusion:**

This is the first randomized controlled trial protocol from the perspective of Qigong connotation to systematically evaluate the effects and relevant molecular mechanism of *Imaginary Working Qigong* for the mental health of a college student population. If our study demonstrates a significant intervention effect, this would provide preliminary higher-quality evidence and establish a further guidance for the application of *Imaginary Working Qigong* program among a college student population.

**Ethics and dissemination:**

The study protocol and consent forms have been approved by the medical and animal experiment ethic committee of BUCM (approval number: BJZYYDX-LL2014005).

## 1 Introduction

Health is understood to be not just the absence of illness, but its definition implies a comprehensive and integrative understanding of people that includes many interrelated social, psychological, and physical factors.^[1]^ Likewise, the World Health Organization (WHO) proposes that^[2]^ the promotion of mental health and the prevention of mental disorders can help to maintain or improve health, have a positive effect on quality of life, and can be economically beneficial. College life is a critical period during which individuals begin to take definitive steps toward independence and is also considered to be the first major transition period of growth and development that bridges adolescence and adulthood.^[3,4]^ The lifestyle and behaviors that an individual develops during this stage may remain into adulthood and impact future health status.^[5–7]^ College students are special populations that are particularly prone to have significantly a high level of psychological distress than their community peers.^[8–10]^ This is due to the fact that university students face multiple stressors such as academic load, constant pressure to succeed, competition with peers, financial burden, teacher or parental pressure, as well as concerns about the future.^[11–13]^ In terms of the psychological health complaints and symptoms, mental health issues are increasing in severity and number on college campuses.^[14,15]^ College students generally have 2 ways to seek help under circumstances of mental health issues: the nonprofessional approach, including asking for help from family members, friends, and so on; and the professional approach, that is, asking for helps from mental health and counseling institutions.^[16,17]^ However, many studies have shown that people hold some stigma toward psychological counseling and believing; consequently, when college students encountered mental health problems, they tended to solve them on their own. Professional helps are the last option on their mind.^[16–19]^ Thus, seeking alternative and complementary methods for management of their symptoms is preferred by many college students with depressive and anxiety disorders or symptoms.

Apparently, the best way to manage stress and mental state is through self-care; in recent years, people have increasingly been using mind-body exercises as complementary and alternative therapies to manage psychological stress.^[20–31]^ Qigong is an ancient psychosomatic discipline, which is part of Traditional Chinese Medicine (TCM). It can be traced back thousands of years, being a highly popular practice nowadays, and is practiced by a large number of people in China.^[32–35]^ Qigong has been explained that Qigong can improve physical fitness, overall well-being, and longevity by achieving a harmonious flow of energy (*Qi*), blood, and fluid throughout the body to relieve pathological stagnation and regulate the function of meridians and visceral organs.^[32–36]^ The practice of Qigong aims to cultivate energy via systematic training exercises, including the coordination of different breathing patterns, rhythmic movements, and meditation, in contrast to conventional exercise.^[27,32,33,37–39]^ Due to its significant promotion of human health and ease of learning,^[27,28,32,33,35,40]^ Qigong is appropriate for nearly anyone of any age, fitness levels, and physical conditions, especially for young college students. Generally, Qigong can be classified into 2 categories: dynamic Qigong (*dong Gong*) and static Qigong (*jing Gong*).^[20,32,33]^ The former involves the coordination of movements and meditation, whereas the latter focuses on mind concentration and body relaxation without physical movement. Qigong is an easily adaptable form of mind-body integrative exercise that can be practiced in anyplace, and anytime, without any special equipment.^[27,41–45]^ It is widely practiced by Chinese not only to improve their physical health but also to control their emotions, manage their stress or depressive/anxiety symptoms, and enhance overall well-being. In recent years, an increased number of studies have documented the effect of qigong on depressive and anxiety symptoms.^[20,21,24,26,27,39,43,46–48]^ Thus, Qigong is a traditional Chinese mind-body exercise, which has the potential as a stress management intervention, as it has all of these key elements described above.

In the current definitions of Qigong,^[49]^*Qigong is the skill of body-mind exercise that integrates the three adjustments of body, breath, mind into “one.”* It was clear that the content of Qigong is based on “the three adjustments” of body, breath, and mind. The aim of the 3 adjustments is to achieve a state of harmonious unity-integrating 3 adjustments into “one,” the state of unity.^[32,49]^ In the process of Qigong training, how to from learning and practicing the 3 adjustments to oneness of 3 adjustments? According to the results of our previous studies, we believe that as long as we enter the state of *Concrete Thinking*, it is relatively easy to achieve this state of Oneness.^[49–55]^ The theory of *Concrete Thinking* comes from the study of the technique of mind adjustment in Chinese Qigong practice.^[49–51]^ The extension and connotation of the concept *in Concrete Thinking* was first accurately defined in *the Thinking Operation in Meditation Practice* edited by Professor Liu Tianjin of Beijing University of Chinese Medicine (BUCM). And *Concrete Thinking* has becomes the basic paradigm of explaining the operation of Qigong.^[49–51]^*Imaginary Working Qigong* (IWQ) not only is a kind of *Mental visualization* (*Cun Xiang*) training method but also is created and developed base on *Concrete Thinking* theory.^[49,56]^ Simply, IWQ is the process of keeping the mind on specific scenery or an imaginary object so that it can visualize it vividly. Attention should be on the difference between IWQ and imagination in daily life. The image from imagination is not very clear—mostly it is an image from the memory. The imaginary object required in IWQ is concrete, with real sensation and perception. Imagining an object belongs to an ideal thinking pattern, and mentally visualizing an object belongs to perceptual thinking patterns in the psychology of thinking. To summarize, adjustment of the mind can make a change in the thinking pattern, which is one of the contents and forms of mental activities. And the purpose of IWQ is ruling out stray thoughts and inducing perceptions. The design and choice of point of focus for IWQ can take the need to induce specific perceptions into account. Although Qigong is practiced in many forms, a gentle and easily practiced form is that of static Qigong. IWQ, as a kind of static Qigong, is a thorough, nonstressful, and low impact form of exercise where participants follow the direction of a trained Qigong teacher, and is commonly and easily performed in group settings. Hence, IWQ is more perception-oriented and can bring about benign sensations of mind and body so as to eliminate stress completely and induce physiological and mental relaxation.^[49,56]^

Our pilot studies have indicated that IWQ program may have effects of tranquilizing, regulating emotion, and managing stress for college students.^[57–59]^ However, there is currently a lack of evidence regarding biological mechanisms underlying the effects of mind adjustment induced IWQ and associations between IWQ and bioelectrical activity of cortical neurons, and change of gene expression in peripheral blood leucocyte, as well as self-reported anxiety, depression, personality, quality of sleep. On this basis, in this study protocol, we will design a randomized control trial (RCT) to systematically evaluate the effects of IWQ on psychological outcomes of the college students, including bioelectrical activity of cortical neurons, as well as self-reported anxiety, depression, personality, quality of sleep, and go a step further to explore and analyze the regarding biological mechanisms underlying the effects of mind adjustment induced IWQ on psychological health in college students and associations between molecules and signal pathways and psychological outcomes of the college students.

## 2 Methods/designs

### 2.1 Study objective and hypotheses

The overall aim of the study will be to examine the feasibility and acceptability of IWQ program among college students. The primary aim of this study will be to describe the protocol for a randomized controlled trial designed to collect preliminary data to systematically ascertain the potential efficacy or improvement effects, and safety of IWQ program as an alternative nonpharmacological intervention on mental health of college students by observing the difference between the IWQ group and unaltered lifestyle control group. The primary hypothesis of this study is that college students who receive a 8-week IWQ training (4-week supervised training and 4-week independence training) intervention will show greater improvement in psychological health than those who keep to their usual daily lifestyle, as assessed immediately after the 4-week supervised training and 4-week independence training intervention, and these benefits will be sustained during 4-week follow-up phase.

The secondary aims of this study are as follows:

(1) To investigate if IWQ is more beneficial to balance effect of brain in college students than usual daily lifestyle.
(2) To adopt objective outcome measures to elucidate the effect of IWQ on specific domains of electroencephalogram and psychological self-rating scales in college students.
(3) To explore molecular biological mechanisms underlying the effects of adjustment of mind-induced IWQ on psychological health in college students based on the change of gene expression tested by CapitalBio Technology Human LncRNA Array v4.

We hypothesis that college students with psychological subhealth state who receive an 8-week IWQ training intervention will achieve greater improvement in specific domains of gene expression and show favorable changes in function of relevant brain regions and mental health than those who maintain their usual daily lifestyle. And adjustment of mind-induced IWQ that originates from synchronization effects of brain active tasks so as to inducing the expression of molecular related signal pathways and improving the psychological subhealth state.

### 2.2 Study design

This study involves a single-center, 2-arm RCT with 1 intervention condition, assessor and statistician-blinded, parallel-controlled trial comparing the psychological effects of IWQ in college students with unaltered lifestyle control repeatedly. The design employed is a 2 (condition) × 4 (time) parallel-group design, which is explanatory in nature. A total of 80 eligible college students from the BUCM will be enrolled and randomly assigned to either the IWQ group or control group (unaltered lifestyle) according 1:1 allocation ratio with allocation concealment. The participants in the IWQ group will accept an 8-week IWQ training (4-week supervised training and 4-week independence training), at the same time the others in the control group will maintain their original lifestyle. The study period is 12 weeks, including a 4-week supervised training, 4-week independence training, and a 4-week follow-up with relevant psychological outcomes; measurement will take place at baseline, 5 weeks (at the end of supervised training), 9 weeks (at the end of independence training), and 13 weeks (after the 4-week follow-up period) by the assessors who are not participated in this trial at the Qigong and Human Science Laboratory of BUCM.

**Figure 1:**
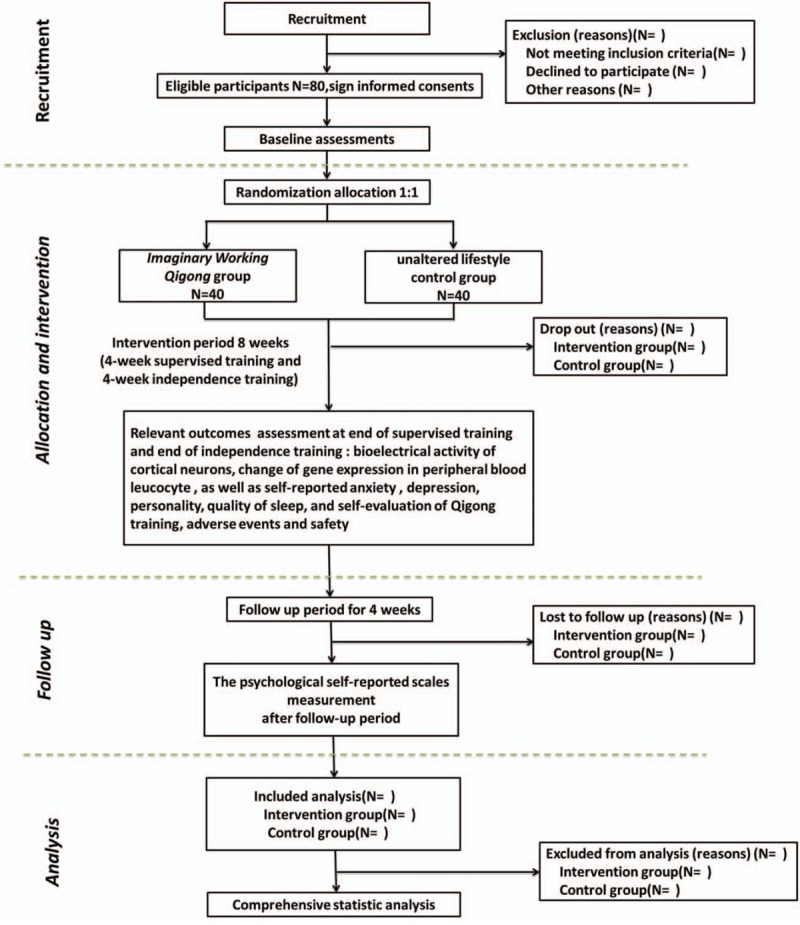
Flow diagram of study design.

The statistical analysis will be performed by special statisticians who are not involved in this trial. An app has been developed for collecting outcome data of relevant psychological self-rating scales and to provide IWQ program intervention in the form of audio content to the participants of the intervention group. The flow diagram for this trial is illustrated in Fig. 1.

### 2.3 Sample size

Out study is a preliminary clinical trial aiming to explore benign effects of IWQ on the psychological well-being of college students from the integrating with electroencephalogram, psychological self-rating scales, and change of gene expression. Therefore, we did not determine sample size basing on statistical calculations and this study is designed as a pilot study to calculate the appropriate sample size for future randomized clinical trials. An alternative approach to estimate the sample sizes for pilot studies with 10 to 40 participants per group was adopted,^[60]^ which is the minimum sample size for evaluating the effect of adjustment of mind-induced IWQ. Accordingly, to exceed the minimal number and combine with the actual situation, a total of 60 participants will be recruited in this research, 30 in each group. Assuming a 20% drop-out rate, we aimed to recruit 80 participants. Each group will require 40 initial participants.

### 2.4 Participant and recruitment

A total of 80 college students from BUCM who are in the first or second year aged 18 to 25 years will be recruited. We plan to advertise the recruitment program of participants through schoolyard media advertisements on the campus bulletin board of BUCM, as well as via campus radio, sending leaflets, and brochures. Interested students will contact the research assistants who will determine eligibility according to the inclusion and exclusion criteria at the recruitment office. The research assistants will explain the purposes and characteristics of the study and ask if they have an interest in participating. The potential participants will be required to sign an informed consent agreement if they fulfill inclusion criteria and not comply with the exclusion criteria before enrollment in this trial. Participants will also be asked to sign a declaration stating that they will participate in training and testing fully to the best of their ability and will continue their involvement in the IWQ program once commenced. A log book will be given to each participant to monitor involvement and practice IWQ as well as any reporting of adverse events (AEs).

### 2.5 Inclusion criteria

Individuals who meet all the following criteria will be included as appropriate participants in the trial:

(1) College students, aged between 18 and 25 years, a full-time freshman or sophomore;
(2) Right-handed;
(3) Voluntarily participate in this study;
(4) Agreeing to participate in this trial after receiving a thorough explanation of the purposes and characteristics of the trial and willing to sign the written informed consent form, available and willing to complete all IWQ training sessions and relevant psychological outcomes assessment on time;
(5) Truthfully filled out the training and testing record forms and cooperate with the relevant psychological outcomes measure.

### 2.6 Exclusion criteria

Participants would be ineligible if they meet any of the following conditions:

(1) Being or having been engaged in a long-term regular exercising meditation Qigong or other forms of Qigong or athletic sports;
(2) Being a member of the Martial Arts Association, Yoga Association, Dance Association, Aerobics Association, Sanda Association, or Taekwondo Association, and so forth;
(3) Those who have a family history of psychosis, neurasthenia, stress disorder, personality disturbance, mental sickness induced by taking psychoactive substances;
(4) Those who have suffered from malignant tumor, severe consumptive disease, cerebrovascular disease, communicable disease, mental illness, and severe cardiovascular, liver, kidney, gastrointestinal and hematological diseases, musculoskeletal system diseases, or other contraindication to mild to moderate physical exertion;
(5) Those who have a high risk of suicide as elicited by interview or have head trauma within the past 6 months.
(6) Those who used antianxietic, antidepressants, antipsychotic drugs or anti-insomnia drugs at a month before the start of the study;
(7) Those who with a metal or heart pacemaker implanted in the body;
(8) Those women who are pregnant, lactating, or planning to become pregnant;
(9) Those who being or having been participated in other similar clinical trials that affect the relevant psychological outcomes of this study.
(10) Those who inability to comprehend and complete the study assessments or to be likely to encounter difficulties in adhering to this study instructions;
(11) Those participants whom the research investigators judge to be inappropriate.

### 2.7 Withdrawal criteria

Participants will request to be withdrawn from the study if they meet any of the following criteria: poor compliance (mean compliance < 85% at the last estimation) or noncompliance, occurrence of a serious AE, initiative exit are unable to progress because of sudden disease, members in the control group have regularly engaged in IWQ.

### 2.8 Randomization and allocation concealment

Randomization will be performed at post-baseline assessment and stratified by age and sex. The random allocation sequence will be produced via the PLAN sentences of IBM Statistic Package for Social Science 21.0 (IBM SPSS 21.0) by a professional, independent data manager who will be not involve in the trial. All the eligible participants will have equal chances of being selected for either IWQ group or unaltered lifestyle control group according to 1:1 equal proportion rule. To make sure that the risk of bias remains low, participants will be registered in the database by means of a participant ID code so that assessors are blinded during the analysis. The random allocation sequence will be managed by a specified project manager who is not involved in the recruitment program of this trail, and be concealed to the screeners and outcome assessors. The assigned results for each participant were sealed in opaque white envelopes. Allocation concealment will be ensured, as the service will not release the randomization code until the participants are recruited into the trial after all baseline measurements are completed. Once participants consented to enter the trial, random assignment envelopes were opened and assignments made to IWQ group or control group in the consecutive order of the participants’ serial numbers. The eligible participants will be informed of the allocation result by the project manager via telephone or e-mail after post-baseline information assessment.

### 2.9 Informed consent

We will fully explain the details of this study to potential participants before they take part in this research, including trial objectives, characteristics, probable benefits, potential risks, as well as the general study process and the responsibilities of both participants and researchers. They will also be informed that their participation in the trial is entirely voluntary and that they can withdraw at any time for any reason. In the event of their withdrawal, the data collected cannot be erased and will be used in the final analyses. All participants who meet all inclusion criteria and none of exclusion criteria and agree with this trial will give their written informed consents before they undergo any interventions related to this study. Their personal information will be always undisclosed and kept confidential. A research assistant will be responsible for obtaining informed consent from all participants.

### 2.10 Blinding

In this study, it is impossible to blind the participants and IWQ coaches due to this being a nonpharmacological intervention trial. Nevertheless, 2 kinds of blind code will be used to blind the outcome assessors and statistician, and we will assign a specified research manager to be in charge of the management of the random allocation sequence and blind code in which the participants’ allocation result will be replaced by using condition “A” or “B.” And the real meaning of “A” or “B” will be signed in the second blind code. First, after close of database, the research manager will deliver the group condition “A” or “B” of participants to the statistician. Second, the research manager will declare the real meaning of group “A” or “B” after analysis of all data is completed. In addition, we will define the obligations of each investigator: the research manager and IWQ coaches will not take part in the assessment of outcome; at the same time, the outcome assessors, data managers, and the statistic analyzer will be not involved in the participants’ screening and allocating. The allocation sequence and blind code will be preserved by an independent project manager who is irrelevant with the recruitment, intervention, assessment, and statistical analysis until the statistical analysis is completed.

### 2.11 Intervention

#### 2.11.1 Imaginary working Qigong group

The IWQ training, includes supervised training and independence training. Supervised training will be performed lasting 4 weeks at the Qigong training classroom of the university and then 4 weeks of independence training according to own situation to decide the time and place. The training scheme originated from the IWQ recorded in *Traditional Chinese Medicine Qigong*^[49]^ (“13th Five-Year” planning teaching materials of China National Higher Education of Chinese Medicine, Beijing: China Press of Traditional Chinese Medicine, chief editor: Tianjin Liu, Wenchun Zhang) and *Chinese Medicine Qigong Training Guidance*^[61]^ (Beijing: China Press of Traditional Chinese Medicine, chief editor: Yulong Wei), which not only is a kind of the traditional Chinese Qigong psychosomatic training method but also is an extension of *Concrete Thinking* theory. IWQ is characterized by translating benign thought subjects into artistic conception by actively processing of the consciousness, and then integrating body and mind into a benign state of psychological harmony, thus regulating bad psychological emotions. Two experienced coaches who have engaged in the IWQ education will teach, correct, and supervise the IWQ training during the whole intervention period.

#### 2.11.2 Control group

No specific exercise training will be administered on the participants in the control group. They will be informed to keep their original daily lifestyle in the intervention period (based on the initial inclusion criteria that they were not exercising on a regular basis) and requested not to commence any relaxation techniques, mindfulness meditation, or any other mindfulness-based training or participate in other regular mind-body exercises, such as yoga or other forms of Qigong. At the completion of the study, they will be given the same 8 weeks of IWQ training after the 13 weeks so as to increase involvement compliance rate.

#### 2.11.3 Intervention regimen

An initial workshop conducted over 10 consecutive half-days by 2 qualified coaches will be designed before IWQ training. Participants received concentrated training: this consisted of instruction in source of IWQ, understanding in the essence, and beneficial mind adjustment effects of IWQ. Participants will be taught the relevant knowledge of IWQ, which includes the rationale that IWQ constitutes a mental health benefit, the action essence of IWQ, the Chinese Medicine Qigong opinion of IWQ. The instruction of IWQ will be both verbal and visual. At the same time, participants will be also obtained information about procedure of this study, and the matters needing attention of individuals in the trial. Participants will be required to learn the key of artistic conception known as “regulating body, breathe, mind” and ancillary exercises with multiple repetitions of IWQ until they get it, as will be confirmed by the professional coaches. The coaches will visit each individual daily to ensure that artistic conception processing is being correctly practiced. Once initial concentrated training was complete, participants were asked to practice supervised training and subsequently independence training. Participants of IWQ group will undergo the regular supervised training together last 4 weeks and then independence training last 4 weeks at a frequency of 5 days per week (from Monday to Friday); training will be performed for 40 minutes per day, and each session will include a warm-up of relaxation for 10 minutes. Specified IWQ was performed and refined for 30 minutes; the practice method is successively as follows: siting, closing eyes, and taking deep breaths; recalling and forming images; artistic conception operation; and mind and body fuse in a benign state of psychological harmony. Specifically, participants are guided through benign state of psychological harmony and artistic conception operation so as to cultivate a benign state of psychological harmony, which involves practicing focusing attention on present-moment sensations in the body without emotionally elaborating on these sensations. Gradually, participants learn to redirect their attention when the mind wanders and to broaden their present-moment awareness to all current internal and external stimuli. Toward the end of training, participants are encouraged to apply this type of awareness to everyday activities. The instructors will visit or supervise each individual to ensure that artistic conception processing is being correctly practiced. All participants will be required to fill self-made *Imaginary Working Qigong* training self-evaluation scale (including self-evaluation of training difficulty, the relaxation of mind and body, breathing control state, the feeling of mind and body during and after the artistic conception operation, level of coordination between body and mind during and after training) and record their daily physical activity information during the intervention period. The compliance of the subjects will be assessed in terms of the number of training attended and the number of training self-evaluation scale per day. In order to exclude bias from the exceed activity of participants, all participants in both groups will be required to record physical activity diaries, including the type and intensity of physical activity or exercises, as well as the sedentary time and sleeping time everyday throughout this study.

### 2.12 Follow-up period

After finishing the training of 8 weeks, follow-up will be done on all participants for 4 weeks. During the 4-week unsupervised follow-up period, all of the participants will return to their original lifestyles, but be required to record their mind and body health condition, daily physical activities, or sport information. The psychological self-reported scales will be re-evaluated at end of follow-up period. All forms will be collected by the data collectors regardless whether they have withdrawn from the trial assessments for reviewing at the end of this trial. The follow-up assessment is designed to evaluate the long-term effects of IWQ on psychological wellbeing of college students.

### 2.13 Outcome assessment

Outcome measurements consist of basic characteristics of information, bioelectrical activity of cortical neurons, and change of gene expression in peripheral blood leucocyte, as well as self-reported anxiety, depression, personality, quality of sleep, and self-evaluation of Qigong training. The basic characteristics of information will be collected at baseline (1–2 weeks before randomized allocation). The relevant primary and secondary outcomes will be assessed at baseline, 5 weeks (at the end of supervised training), 9 weeks (at the end of independence training), and the psychological self-reported scales measurement will be assessed at 13 weeks (after the 4-week follow-up period). Bioelectrical activity of cortical neurons will be assessed at the Qigong and Human Science Laboratory of BUCM by experienced operators and change of gene expression in peripheral blood leucocyte will be detected at the Capitalbio Technology Corporation by professional genetic detection team; all of testing personnel are not otherwise involved in this study. Several outcome assessors who are in charge with the psychological self-reported scales measurement will investigate psychological status of the participants [such as Self-rating Anxiety Scale (SAS),^[62]^ Self-rating Depression Scale (SDS),^[63]^ Eysenck Personality Questionnaire-revised, short Scale for Chinese (EPQ-RSC),^[64,65]^ Pittsburgh Sleep Quality Index (PSQI),^[66]^ Self-made Qigong Training Self-evaluation Scale (SQTSS)] at college student activity center. A summary of all measures in the trial is summarized in Table 1.

**Table 1:**
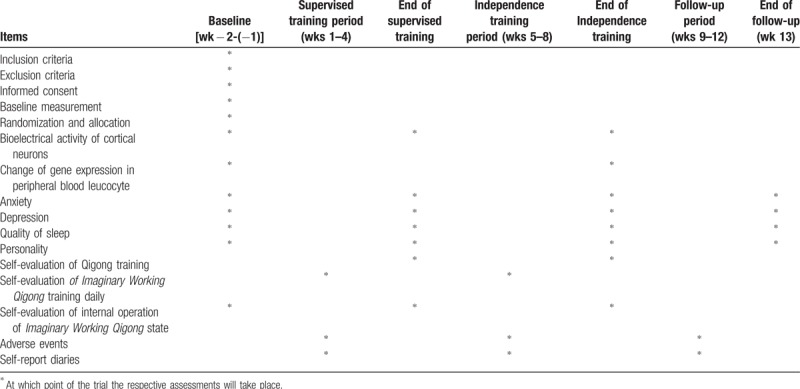
Trial measures processes chart.

#### 2.13.1 Basic characteristics variables

Baseline demographic characteristics information, which could act as covariates or confounds for the tested intervention modality, will be also collected. These include each participant's sex, age, nationality, native place, marital status, handedness, height and weight, medical history, family history of disease, which will be collected by the recruiters using the Self-designed Standardized Questionnaire (SSQ) at baseline. Lifestyle factors, including diet, physical activity or exercise habits, smoking habit, and drinking alcohol habit, will also be recorded. Baseline assessment will be completed before randomization.

#### 2.13.2 Primary outcomes

Primary outcomes consist of bioelectrical activity of cortical neurons, as well as self-reported anxiety and depression.

(1) Bioelectrical activity of cortical neurons, including electroencephalograph power spectrum of δ, θ,α1, α2, β1, β2 in different benign artistic conception of *Imaginary Working Qigong* state, resting state, and natural state that from three aspects of general points, time interval, and different leads. It will be assessed by *the Nuamps 40 channel electroencephalograph (EEG) signal recording and analysis system and the curry-7 acquisition software* produced by Neuroscan Company, at baseline, 5 weeks (at the end of supervised training), and 9 weeks (at the end of independence training).
(2) Anxiety will be measured with SAS. The scale consists of 20 questions that reflect subjective feelings of anxiety symptoms. Participants are asked to respond to each question on a 4-point Likert scale ranging from 0 (never or few time) to 3 (very often or whole time). Higher composite scores indicate greater anxiety.^[62]^
(3) Depression will be measured with SDS. Twenty questions in SDS reflect subjective feelings of depression symptoms. Participants are asked to respond to each question on a 4-point Likert scale ranging from 0 (never or few time) to 3 (very often or whole time). Higher composite scores indicate greater depression.^[63]^

#### 2.13.3 Testing protocol of electroencephalogram

According to the new model of Qigong scientific research “Two-way layout, correlated detection and mutual paraphrase (Two- way layout refers to design both schemes of Qigong internal operation and external detection in the whole design project. Correlated detection refers to detect objective indexes from outside and subjective operational indexes from inside simultaneously during examination. Mutual paraphrase refers to give the meaning of the experimental result by explaining internal and external indexes to each other.)” designed by Professor Liu Tianjin of BUCM. On the contrary, external detection starts only when the Qigong internal operation occurs (participant is asked to click right little finger for signal). In addition, according to the degree of difficulty in benign thought subject processing (artistic conception processing) and whether it can enter the artistic conception quickly, we will use 3 benign thought subjects to operate in the following order: the spring sun is warming my body and mind, I am walking in the rose bushes, every pore of mind is free to breathe. We designed testing scheme of electroencephalogram for IWQ internal operation [such as successively practicing natural relaxation state (I)→six deep breaths (II)→resting state 3 minutess (III)→artistic conception processing: the spring sun is warming my body and mind 1 minute (IV)→resting state 1 minute (V)→artistic conception processing: I’m walking in the rose bushes 1 minute (VI)→resting state 1 minute (VII) →artistic conception processing: every pore of mind is free to breathe 1 minute (VIII) →resting state 1 minute (IX) →natural relaxation state (X)] and external detection. And participants will fill in the self-made internal operation of different benign artistic conception of IWQ state Self-evaluation Scale when the external detection is finished. In addition, different benign artistic conceptions of IWQ internal operations are carried out under the prerecorded instructions and it should be noted that all participants at baseline and participants in the control group just imitating internal operations under the prerecorded instructions.

#### 2.13.4 Secondary outcomes

Change of gene expression in peripheral blood leucocyte, personality, quality of sleep, and self-evaluation of Qigong training were regarded as secondary outcomes.

(1) Change of gene expression in peripheral blood leucocyte including lncRNA and mRNA expression before and after *Imaginary Working Qigong* training. It will be detected by using *CapitalBio Technology Human LncRNA Array v4 designed by* Capitalbio Technology Corporation, China at baseline (1–2 weeks before randomized allocation) and 9 weeks (at the end of independence training).
(2) Personality will be measured using EPQ-RSC revised by Gong Yaoxian and Chen Zhonggeng, etc, which consists of a 48-item self-report scale with four subscales: an extraversion/introversion scale (lower scores 4 more introversion), a neuroticism/stability scale (higher scores indicate greater emotional instability), a psychoticism/socialization scale (higher scores indicate being more reclusive, indifferent to others, and intransigent), and a lie scale. Only the first 3 personality traits were included in our analysis. The lie scale was used to assess the acceptability of respondents’ answers. Participants provided a yes or no response, and responses are scored 1or 0, respectively, except for some reverse-scored items. These 4 traits have been shown to have good factorial similarity across 34 countries in a study that analyzed gender-specific data. In the current sample, Cronbach alpha coefficient for the EPQ-RSC ranged from 0.62 to 0.72.^[64,65]^
(3) Quality of sleep will be indexed by PSQI.^[66]^ The locally validated Chinese version of the PSQI is a self-report questionnaire that assesses multiple dimensions of sleep over the past month. Nineteen individual items generate 7 component scores: subjective sleep quality, sleep latency, sleep duration, habitual sleep efficiency, sleep disturbances, use of sleeping medication, and daytime dysfunction. The sum of the 7 component scores range from 0 to 21. The sum of the 7 component scores yield 1 global score of subjective sleep quality; higher scores represent poorer subjective sleep quality.
(4) SQTSS will be adopted to make self-evaluation about *Imaginary Working Qigong* training. It has 2 subscales, which assess self-evaluation during and after training (4-week supervised training and 4-week independence training). The Self-made Qigong Training Self-evaluation Scale, from the perspective of participants themselves, provides a lot of information about training quality and effects of *Imaginary Working Qigong* training on physiology, respiratory function, and psychology.

### 2.14 Adverse events collection procedure and safety measurements

As we know, AEs were further divided into 2 types, “Serious” and “Other (not including Serious).” In accordance with its definition of an AE and the definition of Qigong-related AEs in the previous literature, the following definition of Qigong-related AEs will be defined^[49]^: “a variety of undesirable experience or any slightly unfavorable and unintended sign, feeling, symptom, physical and mental changes or disease that participants endure during or after treatment or intervention with Qigong training regardless of causal relationship, but are not serious to the point of affecting normal life and work.” And serious AEs are defined that the event led to serious outcomes such as being life-threatening, permanent damage, require either in-patient hospitalization or the prolongation of hospitalization, results in persistent or significant disability/incapacity or death.^[49]^ Thus serious AEs in the Qigong training refer to Qigong “deviation,” also known as “overrunning” of fire and entrance of demons, or deviation for short, is the serious negative somatic or mental reactions in the course of practicing Qigong. Deviation is represented by functional, psychological, emotional, or behavioral disorders that affect the practitioner's normal life or work and is unlikely to disappear spontaneously. Qigong deviation differs from AE that do not interfere with the activities of daily and will mostly disappear spontaneously or be relieved by proper medical intervention.^[49]^ Unlike other potential medications for both substance use disorder and post-traumatic stress disorder, the likelihood of AEs with IWQ is extraordinarily low. However, any AEs related to IWQ will be reported to the researchers or the project manager and the causality with IWQ training will be analyzed regardless of the investigators’ assessments of causality and the coaches or project managers will provide the corresponding treatment to the participant. All details of AEs (such as headache and distension of head, dizziness or vertigo, tinnitus, stuffiness in the chest and difficulty breathing, heart-pounding or palpitations, shaking in the arms or legs, profuse cold perspiration, intensified and strange ceaseless body and limb movement due to *Qi* disorder, neurasthenia, affective disorder, disorder of self-consciousness, hallucination and paranoia, or psychological stress, and so on) whether expected or not, relatedness to the study, time of occurrence, severity, relationship of duration with intervention, and prognosis, which should be recorded in the specific Case Report Forms (CRFs) during the intervention period. Researchers should take necessary measures to deal with all AEs to ensure the safety of subjects and follow-up the subjects until their physical and psychological conditions come back to normal level. If serious AEs happen, the researchers will report to the chief investigator and ethics committee immediately, who will make a decision on whether the participant needs to withdraw from the trial or the study should be adjusted. The causality between serious AEs and intervention will also be analyzed. Those who suffer serious AEs from our trial will be provided free treatment until they recover.

### 2.15 Data collection and management

All data will be entered electronically. There will be a subject identification code list and each participant will receive a participant study identification number. Confidential documents will be retained in the institution's computer in the laboratory. A password system will be utilized to control access. Back up of data will be kept in locked cabinets. The demographic and baseline characteristic data will be collected by screeners when the participants are recruited. The relevant primary and secondary outcome will be measured by the outcome assessors at baseline, 4 weeks (at the end of supervised training), 9 weeks (at the end of independence training), and 13 weeks (after the 4-week follow-up period). Research assistants will conduct quality control of data collection and be responsible for data entry. The project manager will be responsible for initial data cleaning, identifying, coding, and converting into the proper format for data analysis. All data of these participants will be preserved safely and the personal data will only be used for this study. The data management and the whole process of this study will be closely supervised.

### 2.16 Statistical analysis

In descriptive analysis of the sample, continuous variables will be expressed by using mean and standard deviation for normal distribution, and median and interquartile range for non-normal distribution. Normality will be tested using *Kolmogorov–Smirnov* test. Appropriate transformations will be applied in cases of non-normal distribution. And categorical variables will be represented with frequencies or percentages. For the variables with a normal distribution, statistical comparisons between the groups will be made by using an independent *t* test. If the variables have a non-normal distribution of ordinal level, statistical comparison between groups will be made by using the *Mann–Whitney U* test. Measures with a discrete distribution will be expressed as percentages and analyzed by the *X*^*2*^ or Fisher exact test as appropriate. Subgroup analysis will be performed according to participants’ gender and personality. Analysis of variance (ANOVA) will be used for the repeated measurement data, and *post hoc* comparison between groups at different assessment time points will be conducted using multiple comparisons with adjustment to the type 1 error rate. The primary and secondary outcomes of participants who are randomized and receive at least 1 intervention week will be carried out using the intention-to-treat (ITT) analysis. Per-protocol subject analysis (PPS) of the primary outcomes will include participants who have completed the study without major protocol violations and have a compliance rate of > 85%. We will compare the results of the ITT with that PP analysis to check whether the results are consistent or not. If the statistical results of the ITT and PP population data are the same, the results will be deemed to be reliable; if they are contrary, we intend to adopt the results of the ITT population. Safety will be evaluated by tabulations of AEs, and will be presented with descriptive statistics for each group. A Chi-square test or a Fisher exact test will be used to compare the frequency difference in AEs between the 2 groups, and severe AEs will be listed in detail. To explore potential factors that might influence adherence, a general linear model or logistic regression model will be applied to adjust the confounding influence if necessary. Adherence-related data will be taken from training log records. All statistical tests will be performed using IBM SPSS 21.0 with bilateral inspection. The level of statistical significance is assumed at 2-sided P value <.05. The lncRNA+mRNA array data were analyzed for data summarization, normalization, and quality control by using the Gene Spring software V13.0 ( Agilent Technologies, Santa Clara, CA). To select the differentially expressed genes, we used threshold values of ≥2 and ≤−2-fold change and a Benjamini-Hochberg corrected 2-sided *P* value of .05. The data was Log2 transformed and median centered by genes using the Adjust Data function of CLUSTER 3.0 software and then further analyzed with hierarchical clustering with average linkage. Finally, we performed tree visualization by using Java Treeview (Stanford University School of Medicine, Stanford, CA).

## 3 Ethics issue

The trial will be conducted according to ethical guidelines in human research and Declaration of Helsinki.^[67]^ The study protocol and consent forms have been approved by the medical and animal experiment ethic committee of BUCM (approval number: BJZYYDX-LL2014005). The researcher will explain the benefits and risks of the study to each participant. All participants will provide oral and written informed consent form previous to their participation. And they will have the right to withdraw from this trial at any time or at any condition. The research ethics committee will also be in charge of supervising all procedures carried out in our study, including participants’ recruitment, randomization, intervention, data management, and analysis.

## 4 Dissemination

The study protocol has been registered, and is available on the Chinese Clinical Trial Register website (Registration number: ChiCTR-BON-17010848). Results of this study will be published in peer-reviewed journals and the publications will be on “open access” terms. The research results will also be disseminated to the participants, researchers, health care providers and health care professionals, as well as the general public through courses, presentations, and internet. The investigators will be responsible for the publication of the research to share results with broader scientific community, regardless of the magnitude or direction of effect.

## 5 Discussion

The college years are a developmentally crucial period when students make the transition from late adolescence to emerging adulthood.^[4,68]^ Differences between college students and their noncollege peers are generally understudied, but the available evidence shows that various mental disorders are more frequent in college students than in nonstudent populations of a similar age group due to the multiple stressors and lifestyle changes involved.^[8–10,69,70]^ If left ignored and untreated, all of these may lead to students dropping out of college, attempting or committing suicide, or engaging in other risky, dangerous behaviors.^[71–73]^ Because of traditional beliefs and attitudes to mental problems among general population in many societies, people who seek for psychological counseling will not be welcomed or accepted by peers and the society; thus, it is estimated that only a minority of college students with mental health problems seek and receive adequate help.^[16–19]^

In light of this situation, self-care is seen to be the best method to manage stress and mental state among college students with mental disorders. Compared with conventional psychotherapy or exercise therapy, the characteristic of Qigong is self-directed and self-healing, which is designed to improve holistic health and to facilitate mind–body integration.^[34,35,74,75]^ Qigong refers to the cultivation or enhancement of *Qi*. According to TCM, *Qi* is an energy that sustains human well-being and assists in healing Qigong originates from ancient healing and medical practices in Asia and dates back more than 5000 years. It comprises a series of orchestrated practices, including body postures such as standing or sitting, the performance of a range of simple movements, breath practice to accompany the postures or movements, and meditation to achieve a focused state of relaxed awareness. All of these practices are designed to enhance the function of *Qi* through the attainment of deeply focused and relaxed states.^[32–36]^

It is important to acknowledge that the content of Qigong is based on “the three adjustments” of body, breath, and mind. And the aim of the 3 adjustments is to achieve a state of harmonious unity—integrating 3 adjustments into 1.^[34,49]^ Therefore, the key is to understand the practical significance of “adjustment.” On the contrary, it can be seen that every aspect of the 3 adjustments is closely related to “adjustment.” The state of unity of three adjustments is also due to “adjustment,” which can be combined organically, not separated independently. In conclusion, “adjustment” relies mainly on mind, and mind adjusts the process of directing your thoughts and mind; this changes the manner and contents of everyday thinking for the purpose of entering the Qigong state. Thus, mind adjustment is the core of Qigong and it runs through the whole Qigong training and influences within the different contents, stages, and levels of Qigong training. Professor Liu Tianjun definition of *Concrete Thinking* is the procedure that individual purposefully processes (construct, operate, and distinguish) the object image in his consciousness.^[49–51]^ The initiation of somatopsychic experiences is a sign of successful concrete thinking operation.^[49–55]^ Therefore, we can believe that concrete thinking is a basic normal form that explains mind adjustment of Qigong, and as long as enter the state of *Concrete Thinking*, it is relatively easy to achieve the state of Oneness of 3 adjustments.

IWQ is an extension of *Concrete Thinking* theory and through active processing of the consciousness, the benign thought subject translation into the artistic conception, and then makes the body and mind fuse in benign state of psychological harmony, so as to adjust the bad psychological emotions.^[49,56]^ IWQ is embodied into 2 aspects: one is the process of psychological operations, namely, the external landscape, combined with their own needs, both affective, and emotional and idealized, fully conscious processing process, this is a personalized process. Second is the result of the mental adjustment, namely through the process of psychological operations, forming the mental state that conforms to psychological need, base on the process of psychological operations. The former is the foundation of the latter, the latter is the result of the former, but both have a premise, that is the process and the results need to have trigger conditions. This condition is the need of individual psychological state, whose nature because of different reasons such as active or passive, optimistic or pessimistic, benign or malignant determines the process of extension, the nature of the result, and the influence and role in human psychology is also different. Our preliminary results have suggested that Imaginary Working may have shown beneficial effects on decreasing levels of distress and increasing positive mood so as to help modify individuals’ stress responses.^[49,56]^

In this trial, we will apply the *Nuamps 40 channel EEG signal recording and analysis system* to synchronously measure mind adjustment of IWQ and correlation analysis of beneficial effects of IWQ on brain function, and further compares the difference between IWQ state and natural relaxation state. Furthermore, we will deeply explore molecular biological mechanisms underlying the effects of adjustment of mind-induced IWQ on psychological health in college students based on the change of gene expression tested by CapitalBio Technology Human LncRNA Array v4 and analyze the influence of *Imaginary Working Qigong* on college students with different sex and personality. What is more, relevance between bioelectrical activity of cortical neurons and change of gene expression in peripheral blood leucocyte, as well as self-reported anxiety, depression, personality, quality of sleep will be also to observe and explore. This trial focuses on a college student population. Through an 8-week IWQ training (4-week supervised training and 4-week independence training), results from a range of primary and secondary outcome measures will provide the clear information about difference in psychological outcomes and molecular mechanism of mind adjustment between IWQ and usual unaltered lifestyle control group. In this trial, we scrupulously performed the rigorous randomized, parallel-controlled, assessor-blinded, and statistician-blinded design with an appropriate sample (n = 80) to evaluate the effectiveness and safety, feasibility of IWQ on the mental health of college students. We will arrange 2 qualified teachers to serve as the coaches in order to ensure the standardized intervention training for participants. Participants in IWQ group will be gathered to do the exercise at a fixed setting and time and required to fill self-made IWQ training self-evaluation scale. To control the trial bias, all participants in both groups will be required to record their physical activity and sport diary as well as the sedentary time and sleeping time everyday throughout this study. Furthermore, the result evaluators and statistical analysts will be blinded to ensure the authenticity and objectivity of the trial results.

Initially, one of strengths of this study is that the psychological outcomes assessment on several levels involving bioelectrical activity of cortical neurons, and change of gene expression in peripheral blood leucocyte, as well as self-reported anxiety, depression, personality, quality of sleep will make it possible to explore the mechanism of action of IWQ, which offer a comprehensive understanding on the mind adjustment, allowing the researchers to investigate the relationship between subjective scales and objective indicators. At the same time, using the SQTSS to appraise training quality and effects of Qigong training on physiology, respiratory function, and psychology from the perspective of participants themselves is also the unique highlight of our study.

As this is a feasibility study, there are limitations to this study protocol that should be addressed in a full RCT in future. Ideally, everyone involved in an RCT should be blinded, but this is not always feasible in the nonpharmacological trials^[76]^; therefore, performance bias may be inevitable. Although the participants and exercise coaches are not blinded and the psychological outcome measures are self-reported to the participants, the development of some form of sham intervention for use in future studies of Qigong is a desirable goal, and we will make every effort to ensure that outcome assessors, laboratory technicians, data managers, and statisticians are unaware of the treatment allocations and train the whole research team, as well as inform the participants about the details of self-report scales. Second, all participants will come from one and the same university, which may decrease the sample representativeness. Third, limitations of this study are the relatively small sample size (only 80 participants) because the sample size calculation process is not applied in this study. Thus, further research will improve representativeness of the sample by expanding sample diversity.

In summary, this is the first RCT protocol from the perspective of Qigong connotation to systematically evaluate the effects and relevant molecular mechanism of IWQ for the mental health of a college student population. If our study demonstrates a significant intervention effect, this would provide preliminary higher-quality evidence and establish a further guidance for the application of IWQ program among a college student population.

## Acknowledgments

The authors thank Qigong and Human Science Laboratory of BUCM and Ovation Health Science and Technology Co. Ltd, ENN Group for equipment available for this study as well as express gratitude for all participants and researchers who participated in this trial for their cooperation. The authors also would like to deeply acknowledge Professor Tianjin Liu from BUCM and Qing Tang, Tongju Li from Ovation Health Science and Technology Co. Ltd, ENN Group for providing valuable suggestions to conduct this study.

## Author contributions

**Conceptualization:** Yu Guo, Mingmin Xu, Meiqi Ji, Yulong Wei.

**Data curation:** Jialei Zhang, Jian Yan, Yue Chen, Xiaoqian Shao, Jian Yan.

**Formal analysis:** Ying Wang, Jiamei Guo.

**Investigation:** Yu Guo, Mingmin Xu, Meiqi Ji, Zeren Wei.

**Methodology:** Yu Guo, Mingmin Xu, Yulong Wei.

**Project administration:** Yu Guo, Mingmin Xu, Jialei Zhang, Yulong Wei.

**Supervision:** Yu Guo, Yulong Wei, Mingmin Xu.

**Validation:** Yu Guo, Mingmin Xu, Qingchuan Hu, Jiaxuan Lu.

**Visualization:** Yu Guo, Mingmin Xu, Meiqi Ji, Zeren Wei, Jialei Zhang, Qingchuan Hu, Jian Yan, Yue Chen, Jiaxuan Lyu, Xiaoqian Shao, Ying Wang, Jiamei Guo, Yulong Wei, Jiaxuan Lu, Ying Wang.

**Writing – original draft:** Yu Guo, Mingmin Xu.

**Writing – review & editing:** Meiqi Ji, Qingchuan Hu, Jiaxuan Lyu, Yulong Wei, Jiaxuan Lu.

Yu Guo orcid: 0000-0002-1752-1254.
